# Lung Mechanics of Mechanically Ventilated Patients With COVID-19: Analytics With High-Granularity Ventilator Waveform Data

**DOI:** 10.3389/fmed.2020.00541

**Published:** 2020-08-21

**Authors:** Huiqing Ge, Qing Pan, Yong Zhou, Peifeng Xu, Lingwei Zhang, Junli Zhang, Jun Yi, Changming Yang, Yuhan Zhou, Limin Liu, Zhongheng Zhang

**Affiliations:** ^1^Department of Respiratory Care, Sir Run Run Shaw Hospital, Zhejiang University School of Medicine, Hangzhou, China; ^2^College of Information Engineering, Zhejiang University of Technology, Hangzhou, China; ^3^Department of Pulmonary Disease, Sir Run Run Shaw Hospital, Zhejiang University School of Medicine, Hangzhou, China; ^4^Department of Infectious Diseases, Sir Run Run Shaw Hospital, Zhejiang University School of Medicine, Hangzhou, China; ^5^Thoracic Cardiovascular Surgery, Jingmen First People's Hospital, Jingmen, China; ^6^Department of Anesthesiology, The First People's of Hospital of Jingmen City, Jingmen, China; ^7^Department of Administration, Sir Run Run Shaw Hospital, Zhejiang University School of Medicine, Hangzhou, China; ^8^Department of Emergency Medicine, Sir Run Run Shaw Hospital, Zhejiang University School of Medicine, Hangzhou, China

**Keywords:** COVID-19, lung mechanics, mechanical ventilation, asynchrony, asynchonized, prone positioning

## Abstract

**Background:** Lung mechanics during invasive mechanical ventilation (IMV) for both prognostic and therapeutic implications; however, the full trajectory lung mechanics has never been described for novel coronavirus disease 2019 (COVID-19) patients requiring IMV. The study aimed to describe the full trajectory of lung mechanics of mechanically ventilated COVID-19 patients. The clinical and ventilator setting that can influence patient-ventilator asynchrony (PVA) and compliance were explored. Post-extubation spirometry test was performed to assess the pulmonary function after COVID-19 induced ARDS.

**Methods:** This was a retrospective study conducted in a tertiary care hospital. All patients with IMV due to COVID-19 induced ARDS were included. High-granularity ventilator waveforms were analyzed with deep learning algorithm to obtain PVAs. Asynchrony index (AI) was calculated as the number of asynchronous events divided by the number of ventilator cycles and wasted efforts. Mortality was recorded as the vital status on hospital discharge.

**Results:** A total of 3,923,450 respiratory cycles in 2,778 h were analyzed (average: 24 cycles/min) for seven patients. Higher plateau pressure (Coefficient: −0.90; 95% CI: −1.02 to −0.78) and neuromuscular blockades (Coefficient: −6.54; 95% CI: −9.92 to −3.16) were associated with lower AI. Survivors showed increasing compliance over time, whereas non-survivors showed persistently low compliance. Recruitment maneuver was not able to improve lung compliance. Patients were on supine position in 1,422 h (51%), followed by prone positioning (499 h, 18%), left positioning (453 h, 16%), and right positioning (404 h, 15%). As compared with supine positioning, prone positioning was associated with 2.31 ml/cmH_2_O (95% CI: 1.75 to 2.86; *p* < 0.001) increase in lung compliance. Spirometry tests showed that pulmonary functions were reduced to one third of the predicted values after extubation.

**Conclusions:** The study for the first time described full trajectory of lung mechanics of patients with COVID-19. The result showed that prone positioning was associated with improved compliance; higher plateau pressure and use of neuromuscular blockades were associated with lower risk of AI.

## Introduction

The novel coronavirus disease 2019 (COVID-19) imposes an important and urgent threat to global health (1, 2). A substantial proportion of COVID-19 cases will develop severe acute respiratory distress syndrome (ARDS) that requires invasive mechanical ventilation (IMV). The mortality rate of such patients can be as high as 40% (3), depending on comorbidities and the available medical resources. Mechanical ventilation is an important strategy to treat such patients; and lung mechanics can have both prognostic and therapeutic implications. Lung compliance is an important mechanical parameter that should be monitored during IMV. For example, lung recruitment maneuver (RM) has been used to improve lung compliance in severe ARDS (4). There is also evidence in general ARDS population that poor lung compliance without improvement during IMV is associated with poor clinical outcome (5). Patient ventilator asynchrony (PVA) is another important parameter that should be stressed during IMV. Risk factors of PVA has been widely investigated, including hours of the day, use of sedatives, ventilation mode and tidal volume (6, 7). While several studies showed that PVA was associated with clinical outcome, others did not (8, 9). There is preliminary opinion suggesting that lung mechanics of COVID-19 induced ARDS can be quite different from general ARDS (10). However, there is no empirical data on the lung mechanics in COVID-19 patients on IMV. Furthermore, previous studies are limited in several aspects. First, there is no continuous pulmonary mechanics evaluation, including the response of lung recruitment during IMV, all events during prone ventilation. Second, most techniques for the detection of PVA and other parameters requires physical presence of an expert physician at the bedside and is thus only feasible during short periods (11–13). In addition, most studies explored risk factors for PVA in a fixed-time model (14). In reality, both risk factors and PVA and compliance were time-varying (15).

In order to make this gap end, the purpose of the study were 4-folds: (1) to describe the lung mechanics of COVID-19 patients by analyzing high-granularity ventilator waveform data; (2) to explore whether the lung compliance can be influenced by clinical factors, such as recruitment maneuver (RM) and body positioning; (3) to identify risk factors for PVA during IMV in COVID-19 patients; and (4) To describe post-extubation lung functions for survivors with spirometry test.

## Methods

### Study Design and Setting

The study was conducted in the First People's hospital of Jingmen. Clinical data and ventilator wave data were retrospectively collected. All ventilator parameters were collected as longitudinally in hourly basis using a ventilator information system (RespCare™, ZhiRuiSi Tech. Co., Ltd., Hangzhou, China). The impact of RM and positioning on lung compliance was explored in mixed linear model. The study was approved by the ethics committee of the First People's hospital of Jingmen (Approval number: 202002007) and the ethics committee of Sir Run Run Shaw hospital (20200407-32). Individual patient data were de-identified before analysis. Informed consent was waived as determined by the IRB due to retrospective nature of the study design.

### Participants

All COVID-19 patients treated with IMV were included for analysis. COVID-19 was confirmed by one of the following criteria: (1) novel coronavirus nucleic acid was positive as confirmed by real time (RT)-PCT in respiratory or blood specimen; and (2) genetic sequencing showed highly homogenous sequence with the known novel coronavirus (16). For adults with COVID-19 and acute hypoxemic respiratory failure despite conventional oxygen therapy (<92%), we would start using high-flow nasal cannula (HFNC) or non-invasive ventilation (NIV). If the condition further deteriorated and the oxygenation saturation could not be maintained above 92% with HFNC or NIV, IMV would be started (17). Patients were excluded if (1) they were younger than 18 years old; (2) patients with do-not-resuscitate order and (3) with terminally ill disease; (4) patients with incomplete record of waveform data.

### Variables

Demographic data including age and sex were collected as time-fixed data. Hospital mortality was obtained on discharge. Pulmonary functions including forced vital capacity (FVC), forced expiratory volume (FEV1), FEV1/FVC ratio, peak expiratory flow (PEF), Peak inspiratory flow (PIF), maximal inspiratory pressure (MIP), maximal expiratory pressure (MEP) were measured for hospital survivors.

Ventilator parameters including lung compliance, measured PEEP, plateau pressure, tidal volume, work of breathing (WOB), and peak flow rate were measured based on pressure and flow waveforms. Details of the measurement approaches are described in the ESM.

Interventions including RM, positioning, sedatives and neuromuscular blockades were recorded in our analysis. Date and time of these interventions used to match to a period when ventilator parameters and lung mechanics were recorded. The body position was recorded as one of supine, right, left and prone positions at a specific time. Non-supine position was applied during daytime, and the specific positioning (prone, right or left) was determine at the discretion of the attending physician and respiratory therapist depending on the improvement in oxygenation. Prone positioning was applied for at least 10 h one day. RM could be accurately identified from ventilator waves as those with more than 30 cmH_2_O sustained inflation maintained for at least 30 s, the upper limit pressure was 45 cmH_2_O (18).

### Identification of DT and IEE

We developed an interpretable deep learning approach to detect double triggering (DT) and ineffective inspiratory effort during expiration (IEE). Individual deep learning models were developed under all ventilation modes. Under each ventilation mode, two models were established for detecting DT and IEE. Each model uses the raw ventilator waveforms (airway pressure and flow) as input for a binary classification (PVA or non-PVA). It is also capable of explaining the classification by highlighting the segments that contributes mostly to the results. Datasets were annotated by a group of clinical professionals for training and validating the models based on our previously proposed approach (19). The accuracy reached above 95% for both types of PVA in all the ventilation modes. Asynchrony index (AI) was calculated as the number of asynchronous events divided by the number of ventilator cycles and wasted efforts (14). Details of the algorithm development is described in the Electronic Supplemental Material.

### Statistical Analysis

Ventilator parameters were described for each individual patient by median and interquartile range (IQR) (20). Temporal trends of ventilator parameters were visualized with scatter plots and described with Locally Weighted Scatterplot Smoothing (LOWESS) curves (21). These curves were drawn for each individual patient and survivors and non-survivors were denoted with different colors.

Risk factors for IEE and DT were explored with mixed negative binomial regression models, which was a generalization of the Poisson regression allowing for the conditional variance exceeds the conditional mean (22). Random-effects was allowed for intercepts to account for between-subject variance. Predictors of IEE and DT included compliance, plateau pressure, PEEP, TV, respiratory rate, peak flow rate, WOB, sedatives, and neuromuscular blockades. We reported relative risk (RR) for the risk estimate associated with a unit change of these predictors. Risk factors for AI was explored with mixed linear effect model because the response variable AI was in linear scale. We reported coefficient and 95% confidence interval (CI) to represent how AI increased with a unit change in predictors. Factors that can influence lung compliance was explored with a mixed-effects linear model. Factors including age, sex, RM, PEEP, AI, and body position were included in the model. All statistical analyses were performed with RStudio (Version 1.1.463). A two-tailed *p* < 0.05 were considered as statistical significance.

## Results

### Participants and Descriptive Analysis

**Figure 1:**
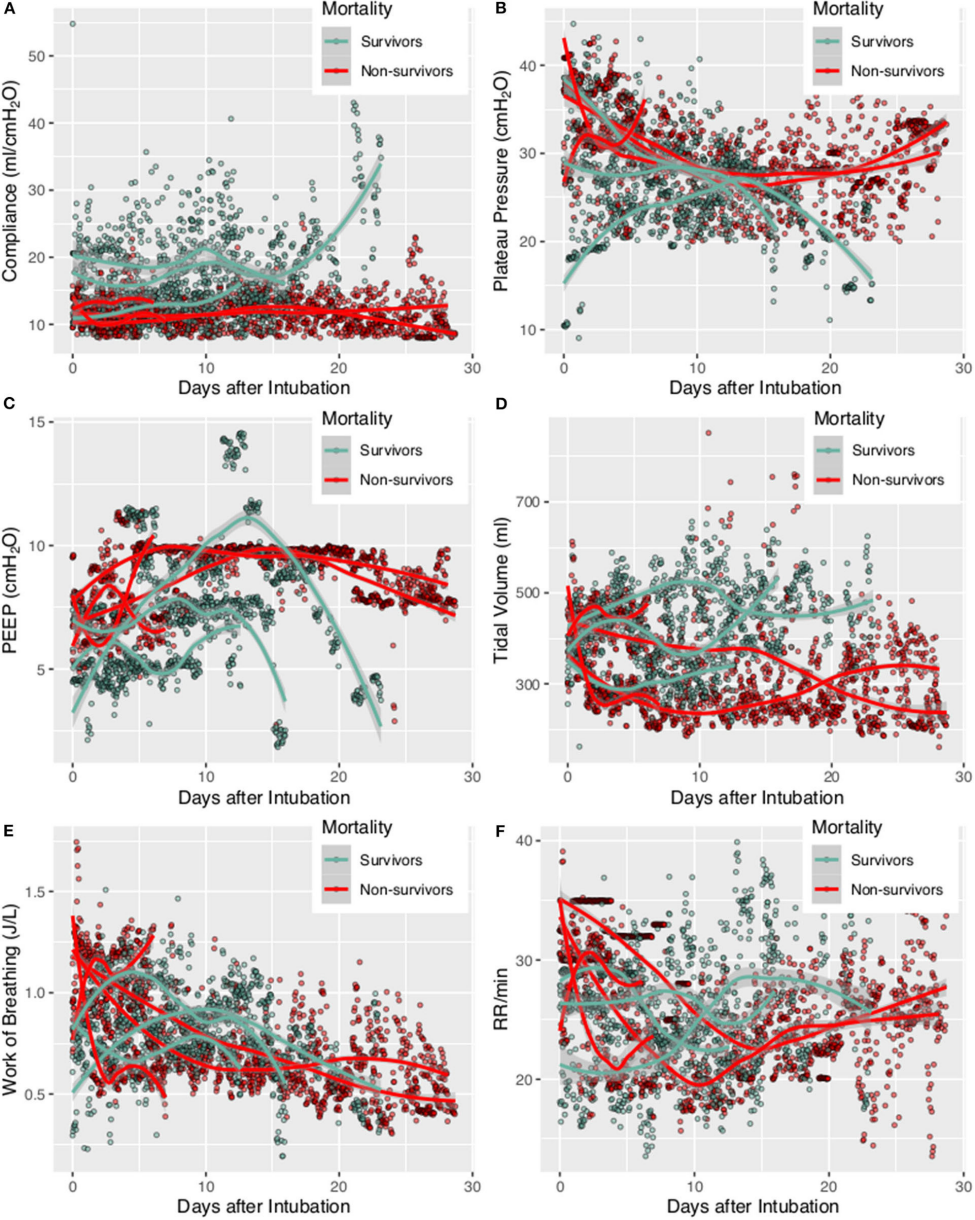
Trajectories of lung mechanics in survivors and non-survivors. The scatter points were smoothed with Locally Weighted Scatterplot Smoothing method. **(A)** Lung compliance was higher in survivors than in non-survivors. **(B)** Plateau pressure of non-survivors followed a U-shaped curve. **(C)** PEEP followed a N-shaped curve with high values during the middle period. **(D)** Tidal volume was higher in survivors, probably due to better lung compliance. **(E)** Consistently decreasing work of breathing was observed in non-survivors. **(F)** Respiratory rate was higher at the beginning, declined rapidly during treatment and reach a nadir at 10–15 days. The respiratory rate was stabilized thereafter at 20–25 per min.

**Figure 2:**
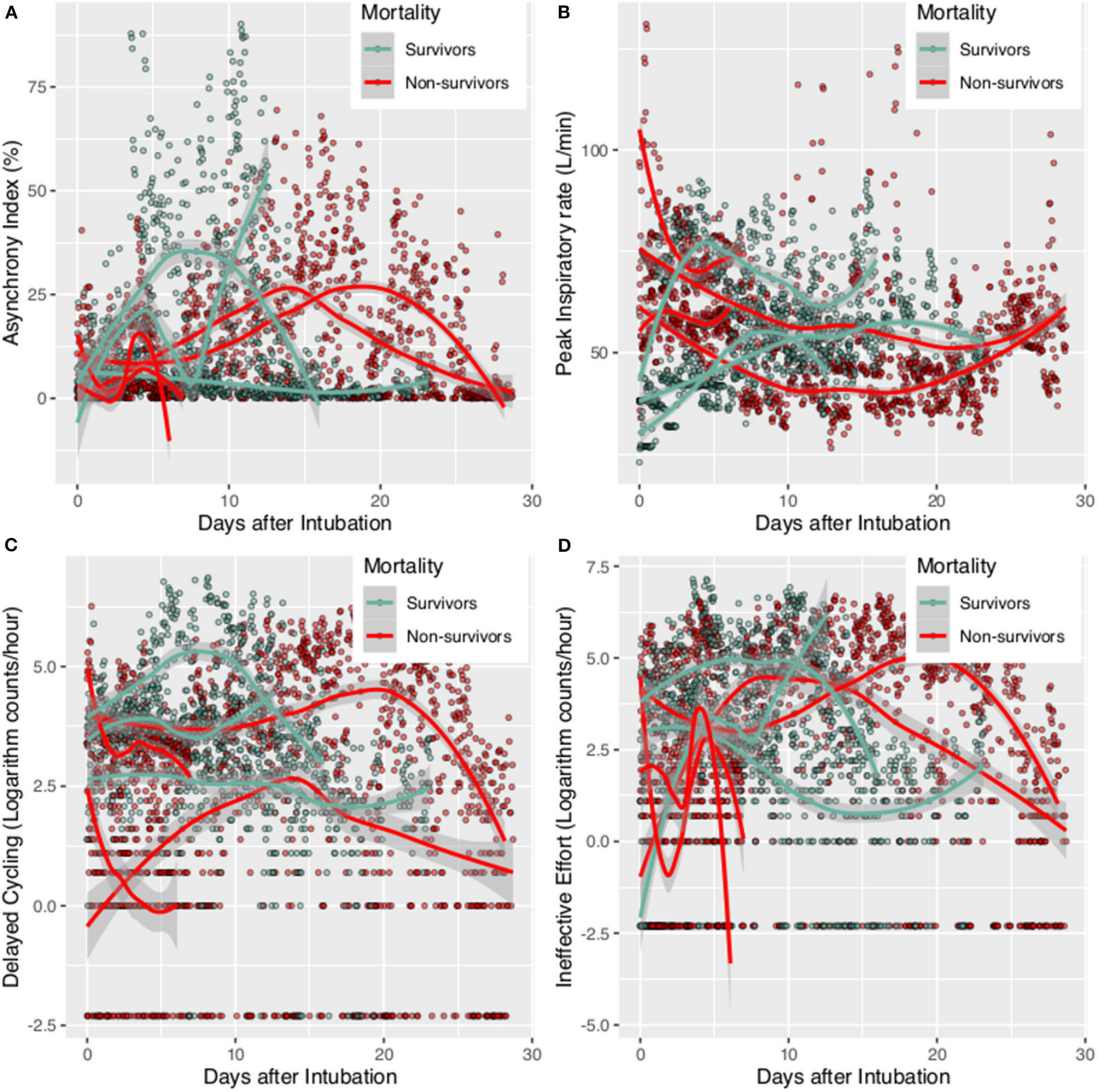
Patient-ventilator Asynchrony during mechanical ventilation for survivors and non-survivors. The scatter points were smoothed with Locally Weighted Scatterplot Smoothing method. **(A)** Asynchrony index trajectory for individual patients; **(B)** Peak inspiratory rate for individual patient; **(C)** delayed cycling for individual patient; **(D)** Ineffective Effort for individual patient.

A total of 7 patients with full record of ventilator waveforms were included for analysis. There was no excluded patient due to predefined exclusion criteria. Four patients died and three survived to hospital discharge (Table 1). A total of 3,923,450 respiratory cycles in 2,778 h were analyzed (average: 24 cycles/min) for the seven patients. Demographics and ventilator parameters were described in Table 1. Due to the limited number of patients, statistical inference was not performed for patient level data. Survivors showed significantly higher lung compliance [15.41 (12.26, 20.85) vs. 11.19 (9.77, 12.8) ml/cmH_2_O; *p* < 0.001], lower PEEP [7.15 (5.14, 8.6) vs. 9.09 (7.68, 9.79) cmH_2_O; *p* < 0.001] and plateau pressure [26.46 (23, 29.23) vs. 30.26 (27.3, 33.1); *p* < 0.001] than non-survivors. Survivors were more likely to adopt prone position than non-survivors (21 vs. 16%; *p* < 0.001). All RM was performed in non-survivors. More neuromuscular blockades were used in non-survivors (Table 2). Patients were on supine position in 1,422 h (51%), followed by prone positioning (499 h, 18%), left positioning (453 h, 16%), and right positioning (404 h, 15%). Survivors showed increasing compliance over time, whereas non-survivors showed persistently low compliance (Figure 1A). Plateau pressure, PEEP and tidal volume are shown in Figures 1B–D. WOB and respiratory rate did not show difference between survivors and non-survivors in temporal pattern (Figures 1E,F). Temporal trends of PVA were not different between survivors and non-survivors (Figure 2).

**Table 1:** Clinical characteristics and lung mechanics of individual subject.

**Variables**	**Patient 1**	**Patient 2**	**Patient 3**	**Patient 4**	**Patient 5**	**Patient 6**	**Patient 7**
Age (years)	57	57	66	81	68	68	54
Sex	Male	Female	Female	Female	Male	Female	Male
Hours from hospital admission to intubation	45	18	163	95	0	116	37
Comorbidities	None	None	Hypertension; diabetes; hepatitis	Hypertension; stroke	Hypertension; diabetes	None	None
Recruitment maneuver (counts)	4	1	0	0	0	0	0
Ejection fraction	58%	61%	58%	60%	50%	NA	NA
Pro-BNP (pg/ml)	521.3	377.1	125.7	1531.0	690.6	687.5	1313.0
CRP (MG/dl)	97.8	16.2	14.6	9.7	88.2	42.3	121.5
CK (U/L)	49.5	154.9	53.0	91.1	119.9	46.8	196.7
CK-MB (U/L)	7.6	18.2	9.6	6.3	9.6	8.2	13.4
LDH (IU/L)	508.5	401.6	453.2	499.4	494.5	482.0	562.8
Troponin T (ng/L)	16.11	16.29	5.50	39.09	38.37	18.88	361.9
Bacteriology (sample/pathogen)	Blood/ *Enterococcus faecium*	Sputum/ *Acinetobacter baumannii*	Negative	Blood/ *Enterococcus faecium*	Negative	Sputum/ *Stenotrophomonas maltophilia*	Sputum/ *Acinetobacter baumannii*
Chest CT involvement	Bilateral	Bilateral	Bilateral	Bilateral	Bilateral	Bilateral	Bilateral
Lesion pattern	Ground glass	Consolidation	Ground glass	Ground glass	Consolidation	Ground glass	Ground glass
Antiviral therapy	Yes	Yes	Yes	Yes	Yes	Yes	Yes
Antibiotics	Yes	Yes	Yes	Yes	Yes	Yes	Yes
AI	6.35 (0.68, 21.89)	14.91 (4.62, 27.59)	5.78 (3.12, 33.97)	1.96 (0.97, 4.23)	6.42 (3.17, 14.26)	0.66 (0.23, 2.72)	21.92 (11.5, 42.38)
Lung compliance (cmH_2_O)	12.15 (10.06, 14.31)	12.17 (10.72, 14.49)	12.45 (11.03, 14.22)	29.04 (23.65, 34.16)	11.24 (9.94, 12.92)	15.74 (14.06, 18.16)	23.2 (17.03, 29.96)
PEEP	8.07 (7.6, 9.82)	9.56 (9.04, 9.82)	5.26 (5.05, 7.03)	8.69 (4.69, 9.51)	7.22 (6.48, 7.86)	7.67 (6.11, 9.39)	7.26 (6.46, 7.66)
Plateau pressure (cmH_2_O)	30.22 (27.95, 33.65)	30.87 (28.05, 34.02)	30.6 (28.35, 35.47)	24 (20.16, 26.01)	31.1 (30.03, 35.56)	32.53 (30.74, 33.84)	28.16 (23.99, 32.66)
Tidal volume (ml)	335.74 (249.1, 405.16)	276.19 (238.69, 329.52)	302.37 (280.24, 339.13)	477.07 (430.38, 523.45)	274.73 (258.55, 292.78)	454.52 (432.26, 474.59)	425.13 (371.11, 474.42)
Respiratory rate (/min)	26.35 (23.4, 31.94)	23.95 (20.09, 28.12)	26.73 (23.45, 28.91)	23.22 (19.86, 29.56)	22.97 (19.97, 27.37)	29.97 (27.92, 30.14)	26.07 (23.39, 28.67)
WOB (J/L)	0.69 (0.51, 0.95)	0.68 (0.6, 0.81)	0.74 (0.68, 0.82)	0.78 (0.67, 0.89)	0.6 (0.57, 0.71)	1.16 (1.05, 1.21)	0.97 (0.78, 1.08)
Peak flow rate (ml/min)	55.69 (47.21, 65.24)	43.35 (37.68, 55.54)	42.22 (40.57, 56.21)	50.25 (44.3, 57.07)	74.94 (68.96, 79.37)	57.83 (56.26, 60.28)	69.95 (58.66, 78.49)
Mortality	Died	Died	Alive	Alive	Died	Died	Alive

*AI, asynchrony index; WOB, work of breathing; PEEP, positive end expiratory pressure; LDH, Lactate Dehydrogenase; CRP, C-reactive protein; BNP, B-type natriuretic peptide; CK, Creatine kinase*.

**Table 2:** Longitudinal variables compared between survivors and non-survivors.

**Variables**	**Total (*n* = 2,778 h)**	**Alive (*n* = 1,160 h)**	**Died (*n* = 1,618 h)**	***p***
Neuromuscular blockades, *n* (%)	81 (3)	23 (2)	58 (4)	0.018
Sedative, *n* (%)	305 (11)	156 (13)	149 (9)	<0.001
Recruitment maneuver, *n* (%)	5 (0)	0 (0)	5 (0)	0.079
Asynchrony Index (%), Median (IQR)	4.95 (1.69, 18.93)	4.84 (2.16, 16.29)	5.07 (1.11, 20.04)	0.007
Compliance, Median (IQR)	12.28 (10.4, 15.22)	15.41 (12.26, 20.85)	11.19 (9.77, 12.8)	<0.001
Position, *n* (%)				<0.001
Prone	499 (18)	248 (21)	251 (16)	
Right	404 (15)	145 (12)	259 (16)	
Left	453 (16)	225 (19)	228 (14)	
Supine	1422 (51)	542 (47)	880 (54)	
Plateau pressure (cmH_2_O), Median (IQR)	28.44 (24.9, 32.17)	26.46 (23, 29.23)	30.26 (27.3, 33.1)	<0.001
PEEP (cmH_2_O), Median (IQR)	7.92 (6.87, 9.64)	7.15 (5.14, 8.6)	9.09 (7.68, 9.79)	<0.001
Tidal volume (ml), Median (IQR)	356.44 (274.71, 445.04)	422.95 (343.21, 487.8)	298.98 (249.24, 390.7)	<0.001
Respiratory rate (/min), Median (IQR)	25.65 (21.91, 29.04)	25.49 (22.03, 28.91)	25.78 (21.83, 29.54)	0.052
WOB, Median (IQR)	0.75 (0.61, 0.94)	0.81 (0.69, 0.94)	0.7 (0.58, 0.94)	<0.001
Peak flow rate (ml/min), Median (IQR)	54.43 (44.08, 64.08)	53.8 (43.48, 63.31)	54.69 (44.75, 64.4)	0.654
DT (/h), Median (IQR)	29 (7, 65)	35 (14, 65)	24 (3, 65.75)	<0.001
IEE (/h), Median (IQR)	29 (4, 153)	21 (5, 100.25)	41 (3, 176)	0.025

*WOB, work of breathing; PEEP, positive end expiratory pressure; DT, delayed triggering; IEE, ineffective effort during expiration; IQR, interquartile range*.

### Factors Associated With PVA

Risk factors for PVA (IEE and DT) were investigated in the mixed negative binomial regression models. Higher plateau pressure (RR: 0.945; 95% CI: 0.934–0.956; *p* < 0.001) and respiratory rate (RR: 0.963; 95% CI: 0.951–0.976; *p* < 0.001) was associated with less IEE. However, greater tidal volume and WOB were associated with more IEE. In contrast to IEE, higher respiratory rate was associated with increased risk of DT (RR: 1.066; 95% CI: 1.054–1.078; *p* < 0.001). Higher plateau pressure (Coefficient: −0.90; 95% CI: −1.02 to −0.78) and neuromuscular blockades (Coefficient: −6.54; 95% CI: −9.92 to −3.16) were associated with lower AI. Sedatives had no significant impact on PVAs (Table 3).

**Table 3:** Mixed negative binomial regression model exploring risk factors for asynchrony.

**Variables**	**RR for IEE (95% CI)**	***p***	**RR for DT (95% CI)**	***p***	**Coefficient for AI (95% CI)**	***p***
Compliance	0.991 (0.977, 1.005)	0.199	1.005 (0.994, 1.017)	0.345	0.17 (0.03, 0.31)	0.016
Plateau pressure	0.945 (0.934, 0.956)	<0.001	0.962 (0.953, 0.972)	<0.001	−0.90 (−1.02, −0.78)	<0.001
PEEP	1.018 (0.982, 1.056)	0.337	1.122 (1.091, 1.154)	<0.001	1.56 (1.23, 1.88)	<0.001
Tidal volume	1.003 (1.002, 1.004)	<0.001	1.003 (1.002, 1.003)	<0.001	0.02 (0.01, 0.02)	<0.001
Respiratory rate	0.963 (0.951, 0.976)	<0.001	1.066 (1.054, 1.078)	<0.001	−0.12 (−0.24, −0.00)	0.049
Peak flow rate	0.996 (0.991, 1.001)	0.082	0.998 (0.993, 1.002)	0.226	−0.07 (−0.11, −0.02)	0.008
WOB	4.066 (2.954, 5.595)	<0.001	2.562 (2.007, 3.272)	<0.001	8.52 (5.80, 11.25)	<0.001
Neuromuscular blockades	0.5 (0.355, 0.704)	<0.001	0.576 (0.434, 0.764)	<0.001	−6.54 (−9.92, −3.16)	<0.001
Sedatives	0.959 (0.797, 1.153)	0.657	1.072 (0.923, 1.246)	0.362	1.33 (−0.49, 3.14)	0.152

*WOB, work of breathing; PEEP, positive end expiratory pressure; DT, delayed triggering; IEE, ineffective effort during expiration; RR, relative risk*.

### Lung Compliance

In multivariable mixed-effects linear model, we found two variables were significantly associated with lung compliance. Each 1 cmH_2_O increase in PEEP was associated 0.27 ml/cmH_2_O decrease in lung compliance (95% CI: −0.36 to −0.18; *p* < 0.001). As compared with supine positioning, prone positioning was associated with 2.31 ml/cmH_2_O (95% CI: 1.75–2.86; *p* < 0.001) increase in lung compliance. Right (coefficient: 1.63; 95% CI: 1.08–2.19 ml/cmH_2_O; *p* < 0.001) and left (coefficient: 0.63; 95% CI: 0.20–1.06 ml/cmH_2_O; *p* = 0.004) positioning were both associated with improve lung compliance (Table 4).

**Table 4:** Mixed linear model exploring factors associated with compliance.

**Variables**	**Coefficient (95% CI)**	***p***
Sex (Female as reference)	5.14 (−9.11, 19.39)	0.334
Recruitment	0.40 (−3.14, 3.94)	0.825
PEEP	−0.27 (−0.36, −0.18)	<0.001
Age (with each year increase)	0.22 (−0.32, 0.76)	0.291
Days from admission to intubation	0.02 (−0.03, 0.07)	0.291
Asynchrony Index (with each 1% increase)	0.01 (−0.00, 0.02)	0.113
Body position (supine as reference)		
Prone	2.31 (1.75, 2.86)	<0.001
Right	1.63 (1.08, 2.19)	<0.001
Left	0.63 (0.20, 1.06)	0.004

*PEEP, positive end expiratory pressure; CI, confidence interval*.

### Spirometry Test for Survivors

Spirometry tests were performed in survivors at day 8, 11, and 13 after extubation. It showed that FVC was consistently decreased for the three measurements. FEV1/FVC was decreased in patient 3 (0.73 at day 8 and 0.707 at day 11); but was preserved in patient 7. PEF, MIP, and MEP were all decreased for the three measurements (Table 5).

**Table 5:** Spirometry tests for survivors after extubation.

	**Patient 3**	**Patient 3**	**Patient 7**
	**(Day 8)**	**(Day 11)**	**(Day 14)**
FVC/predicted FVC	1,223/2,419	1,152/2,419	1,078/3,777
FEV1/predicted FEV1	896/1,849	884/1,849	865/1,789
FEV1/FVC	0.73	0.707	0.850
PEF/predicted PEF	103/350	171/350	65/544
PIF	70	107	58
MIP/predicted MIP	43/71	27/71	15/113
MEP/predicted MEP	43/130	42/130	22/212

*FVC, forced vital capacity; FEV1, forced expiratory volume; FEV1/FVC ratio; PEF, peak expiratory flow; PIF, peak inspiratory flow; MIP, maximal inspiratory pressure; MEP, maximal expiratory pressure*.

## Discussion

The study integrated high-granularity ventilator waveform data with clinical variables to describe the temporal change of lung mechanics of critically ill patients with COVID-19. At the time of intubation, the lung compliance was similar in survivors and non-survivors; but the survivors showed gradually improved compliance. Prone positioning is effective in improve lung compliance. Two types of PVA, IEE, and DT, were identified with deep learning algorithm. Higher plateau pressure and use of muscular relaxant were associated with lower risk of PVAs. Spirometry tests showed that pulmonary functions were significantly compromised after recovery from COVID-19 induced ARDS. Long-term follow up for the change of pulmonary functions would be relevant.

Although the lung compliance was similar at the time of intubation, survivors showed gradual improvement in lung compliance, while non-survivors showed persistently low lung compliance. This is consistent with other studies that lung compliance was an independent predictor of mortality (5, 23, 24). An important finding in our study was that RM was not effective in improving lung compliance, which is in contrast to findings from general ARDS patients. Although the effect of RM on mortality was conflicting in general ARDS, it has been consistently reported to be able to improve lung compliance (25–27). For example, Kung and colleagues observed that the respiratory system compliance was significantly higher in the RM group from day 1 to 7 (25). There is evidence that direct/pulmonary ARDS is more responsive to RM than indirect/extrapulmonary ARDS. While only 21% patients with lower percentage of recruitable lung were caused by pulmonary ARDS, 51% patients with higher percentage of recruitable lung caused by pulmonary ARDS (*p* = 0.01) (28). Thus, COVID-19 induced ARDS is pulmonary ARDS but is less responsive to RM as shown in our study. The second reasons may be due to the fact that we only employed sustained inflation RM. Since there are many types of RM, it is largely unknown whether other types of RM can be effective in improve lung compliance in COVID-19 patients. Finally, the ARDS in COVID-19 may be due to viral, bacterial, or any kind of lung insults. Thus, the RM should not be able to demonstrate the benefits in this group of patients.

PVAs are commonly observed in patients with IMV, especially those with protective ventilation strategy. Our study observed that AI was 4.95% (IQR: 1.69–18.93) in overall observed hours. Non-survivors had more AI than survivors, indicating AI is a risk factor for mortality, which was consistent with other studies (29). Ventilator parameters can have differing effects on different PVA types. For example, while higher respiratory rate was associated with lower risk of IEE, it was associated with higher risk of DT. Use of neuromuscular blockades was associated with lower risk of both IEE and DT. However, we did not observe significant effect of sedatives on AI. Other studies have shown that Propofol or other sedatives can reduce AI (13, 30). It is not surprising to observe that neuromuscular blockades are associated with significantly reduced risk of PVAs.

Post-extubation pulmonary function has never been reported for COVID-19 patients. Our results indicated that pulmonary functions can be significantly compromised in a short period. The FVC is reduced to one third of the predicted value. Other pulmonary function parameters, such as PEF and MIP were also reduced by one third of the predicted value. Boucher and coworkers observed that the pulmonary function can be significantly compromised in pediatric ARDS in short follow-up period (31). In adult patients with general ARDS, the FVC can recover to 3.34 ± 0.77 and 3.78 ± 1.11 L at 1 and 6 months follow up (32), which is significantly higher than that in our study. However, since we did not obtain the long term follow up data, it is largely unknown whether COVID-19 can have long-term effect on pulmonary functions.

Several limitations should be acknowledged in the study. First, the sample size was limited, which prohibited patient-level analysis. The effect of prone-positioning on mortality outcome could not be analyzed with sufficient statistical power. Thus, further large-scale studies are needed to validate our findings. However, our data is rich with high-granularity waveform data, which allows for patient-hour analysis for epidemiological analysis. Second, we only developed deep learning algorithms for identifying two types of PVA. There are other types of PVAs, such as reverse triggering and short/long cycling. However, these analyses are not applicable in pressure-controlled ventilation and pleural pressure is required for reverse triggering (33). Third, the impact of sedative on PVAs were estimated without the dosing of sedatives. We only recorded the use of sedatives as a binary variable. Such treatment would lose some information but is easy to interpret because different sedatives imposes challenge to standardize the dose. Finally, the pulmonary function was measured in a short period of time; long-term follow up data may provide important information for critically ill COVID-19 patients.

## Conclusions

In conclusion, the study for the first time described full trajectory of lung mechanics of patients with COVID-19. The result showed that prone positioning was associated with improved compliance; higher plateau pressure and use of neuromuscular blockades were associated with lower risk of AI. RM was not associated with improvement on compliance.

## Data Availability Statement

The datasets used and/or analyzed during the current study are available from the corresponding author on reasonable request.

## Ethics Statement

The study was approved by the ethics committee of the First People's Hospital of Jingmen (Approval number: 202002007) and the ethics committee of Sir Run Run Shaw hospital (20200407-32). Informed consent was waived as determined by the IRB due to retrospective nature of the study design.

## Author Contributions

HG, QP, and ZZ analyzed and interpreted the results and drafted the manuscript. JZ, CY, and LZ handled the FINNAKI data. All authors took part in designing the study, revised the manuscript critically for important intellectual content, read, and approved the final manuscript.

## Conflict of Interest

The authors declare that the research was conducted in the absence of any commercial or financial relationships that could be construed as a potential conflict of interest.

## Abbreviations

AI: asynchrony index
WOB: work of breathing
PEEP: positive end expiratory pressure
DT: delayed triggering
IEE: ineffective effort during expiration
IQR: interquartile range
COVID-19: coronavirus disease 2019
PVA: patient-ventilator asynchrony
ARDS: acute respiratory distress syndrome
IMV: invasive mechanical ventilation.

## References

[1] Guan W-J Ni Z-Y Hu Y Liang W-H Ou C-Q He J-X Clinical characteristics of coronavirus disease 2019 in China. N Engl J Med. (2020) 382:1708–20. 10.1056/NEJMoa2002032 32109013 PMC7092819

[2] Ki M Task Force for 2019-nCoV. Epidemiologic characteristics of early cases with 2019 novel coronavirus (2019-nCoV) disease in Republic of Korea. Epidemiol Health. (2020) 42:e2020007. 10.4178/epih.e2020007 32035431 PMC7285424

[3] Yang X Yu Y Xu J Shu H Xia J Liu H. Clinical course and outcomes of critically ill patients with SARS-CoV-2 pneumonia in Wuhan, China: a single-centered, retrospective, observational study. Lancet Respir Med. (2020) 8:475–81. 10.1016/S2213-2600(20)30079-5 32105632 PMC7102538

[4] Pensier J de Jong A Hajjej Z Molinari N Carr J Belafia F. Effect of lung recruitment maneuver on oxygenation, physiological parameters and mortality in acute respiratory distress syndrome patients: a systematic review and meta-analysis. Intensive Care Med. (2019) 45:1691–702. 10.1007/s00134-019-05821-9 31701204

[5] Morales-Quinteros L Schultz MJ Bringué J Calfee CS Camprubí M Cremer OL. Estimated dead space fraction and the ventilatory ratio are associated with mortality in early ARDS. Ann Intensive Care. (2019) 9:128. 10.1186/s13613-019-0601-0 31754866 PMC6872683

[6] Ramírez II Adasme RS Arellano DH Rocha ARM Andrade FMD Núñez-Silveira J. Identifying and managing patient-ventilator asynchrony: an international survey. Med Intens. (2019). [Epub ahead of print]. 10.1016/j.medin.2019.09.004. 31668560

[7] Bassuoni AS Elgebaly AS Eldabaa AA Elhafz AAA. Patient-ventilator asynchrony during daily interruption of sedation versus no sedation protocol. Anesth Essays Res. (2012) 6:151–6. 10.4103/0259-1162.108296 25885608 PMC4173465

[8] Blanch L Villagra A Sales B Montanyà J Lucangelo U Luján M. Asynchronies during mechanical ventilation are associated with mortality. Intensive Care Med. (2015) 41:633–41. 10.1007/s00134-015-3692-6 25693449

[9] Vaporidi K Babalis D Chytas A Lilitsis E Kondili E Amargianitakis V. Clusters of ineffective efforts during mechanical ventilation: impact on outcome. Intensive Care Med. (2017) 43:184–91. 10.1007/s00134-016-4593-z 27778044

[10] Gattinoni L Coppola S Cressoni M Busana M Rossi S Chiumello D. Covid-19 does not lead to a “typical” acute respiratory distress syndrome. Am J Respir Crit Care Med. (2020). [Epub ahead of print]. 10.1164/rccm.202003-0817LE. 32228035 PMC7233352

[11] See KC Sahagun J Taculod J. Defining patient-ventilator asynchrony severity according to recurrence. Intensive Care Med. (2020) 32:1515. 10.1007/s00134-020-05974-y 32095850 PMC7223983

[12] De Haro C Ochagavia A López-Aguilar J Fernandez-Gonzalo S Navarra-Ventura G Magrans R. Patient-ventilator asynchronies during mechanical ventilation: current knowledge and research priorities. Intensive Care Med Exp. (2019) 7:43–4. 10.1186/s40635-019-0234-5 31346799 PMC6658621

[13] Conti G Ranieri VM Costa R Garratt C Wighton A Spinazzola G. Effects of dexmedetomidine and propofol on patient-ventilator interaction in difficult-to-wean, mechanically ventilated patients: a prospective, open-label, randomised, multicentre study. Crit Care. (2016) 20:206–8. 10.1186/s13054-016-1386-2 27368279 PMC4930611

[14] Sousa ML de A Magrans R Hayashi FK Blanch L Kacmarek RM Ferreira JC. Predictors of asynchronies during assisted ventilation and its impact on clinical outcomes: the EPISYNC cohort study. J Crit Care. (2020) 57:30–5. 10.1016/j.jcrc.2020.01.023 32032901

[15] Zhang Z Reinikainen J Adeleke KA Pieterse ME Groothuis-Oudshoorn CGM. Time-varying covariates and coefficients in Cox regression models. Ann Transl Med. (2018) 6:121. 10.21037/atm.2018.02.12 29955581 PMC6015946

[16] Jin Y-H Cai L Cheng Z-S Cheng H Deng T Fan Y-P. A rapid advice guideline for the diagnosis and treatment of 2019 novel coronavirus (2019-nCoV) infected pneumonia (standard version). Mil Med Res. (2020) 7:4–23. 10.1186/s40779-020-0233-6 32029004 PMC7003341

[17] Alhazzani W Møller MH Arabi YM Loeb M Gong MN Fan E Surviving sepsis campaign: guidelines on the management of critically ill adults with coronavirus disease 2019 (COVID-19). Intensive Care Med. (2020) 44:1691–34. 10.1007/s00134-020-06022-5 PMC7101866 32222812

[18] Arnal J-M Paquet J Wysocki M Demory D Donati S Granier I. Optimal duration of a sustained inflation recruitment maneuver in ARDS patients. Intensive Care Med. (2011) 37:1588–94. 10.1007/s00134-011-2323-0 21858522

[19] Zhang L Mao K Duan K Fang S Lu Y Gong Q. Detection of patient-ventilator asynchrony from mechanical ventilation waveforms using a two-layer long short-term memory neural network. Comput Biol Med. (2020) 120:103721. 10.1016/j.compbiomed.2020.103721 32250853

[20] Zhang Z. Univariate description and bivariate statistical inference: the first step delving into data. Ann Transl Med. (2016) 4:91. 10.21037/atm.2016.02.11 27047950 PMC4791343

[21] Cleveland WS LOWESS: a program for smoothing scatterplots by robust locally weighted regression. Am. Stat. (1981) 35:54 10.2307/2683591

[22] Kondo Y Zhao Y Petkau J. A flexible mixed-effect negative binomial regression model for detecting unusual increases in MRI lesion counts in individual multiple sclerosis patients. Stat Med. (2015) 34:2165–80. 10.1002/sim.6484 25784219

[23] Kim J-S Kim Y-J Kim M Ryoo SM Sohn CH Ahn S. Impact of lung compliance on neurological outcome in patients with acute respiratory distress syndrome following out-of-hospital cardiac arrest. J Clin Med. (2020) 9:527. 10.3390/jcm9020527 32075160 PMC7073731

[24] Bellani G Grassi A Sosio S Gatti S Kavanagh BP Pesenti A. Driving pressure is associated with outcome during assisted ventilation in acute respiratory distress syndrome. Anesthesiology. (2019) 131:594–604. 10.1097/ALN.0000000000002846 31335543

[25] Kung S-C Hung Y-L Chen W-L Wang C-M Chang H-C Liu W-L. Effects of stepwise lung recruitment maneuvers in patients with early acute respiratory distress syndrome: a prospective, randomized, controlled trial. J Clin Med. (2019) 8:231. 10.3390/jcm8020231 30744214 PMC6406466

[26] Goligher EC Hodgson CL Adhikari NKJ Meade MO Wunsch H Uleryk E. Lung recruitment maneuvers for adult patients with acute respiratory distress syndrome. A systematic review and meta-analysis. Ann Am Thorac Soc. (2017) 14:S304–11. 10.1513/AnnalsATS.201704-340OT 29043837

[27] Spadaro S Mauri T Böhm SH Scaramuzzo G Turrini C Waldmann AD. Variation of poorly ventilated lung units (silent spaces) measured by electrical impedance tomography to dynamically assess recruitment. Crit Care. (2018) 22:26–9. 10.1186/s13054-017-1931-7 29386048 PMC5793388

[28] Gattinoni L Caironi P Cressoni M Chiumello D Ranieri VM Quintel M. Lung recruitment in patients with the acute respiratory distress syndrome. N Engl J Med. (2006) 354:1775–86. 10.1056/NEJMoa052052 16641394

[29] Bruni A Garofalo E Pelaia C Messina A Cammarota G Murabito P. Patient-ventilator asynchrony in adult critically ill patients. Miner Anesthesiol. (2019) 85:676–88. 10.23736/S0375-9393.19.13436-0 30762325

[30] Jakob SM Ruokonen E Grounds RM Sarapohja T Garratt C Pocock SJ Dexmedetomidine vs midazolam or propofol for sedation during prolonged mechanical ventilation: two randomized controlled trials. JAMA. (2012) 307:1151–60. 10.1001/jama.2012.304 22436955

[31] Boucher V Mathy C Lacroix J Émériaud G Jouvet P Tse SM. Post-discharge respiratory outcomes of children with acute respiratory distress syndrome. Pediatr Pulmonol. (2020) 55:468–73. 10.1002/ppul.24581 31765521

[32] Toufen C Jr. De Santis Santiago RR Hirota AS Carvalho ARS Gomes S Amato MBP. Driving pressure and long-term outcomes in moderate/severe acute respiratory distress syndrome. Ann Intensive Care. (2018) 8:119. 10.1186/s13613-018-0469-4 30535520 PMC6286297

[33] Akoumianaki E Lyazidi A Rey N Matamis D Perez-Martinez N Giraud R. Mechanical ventilation-induced reverse-triggered breaths: a frequently unrecognized form of neuromechanical coupling. Chest. (2013) 143:927–38. 10.1378/chest.12-1817 23187649

