# Supplementary material for: Lung Mechanics of Mechanically Ventilated Patients With COVID-19: Analytics With High-Granularity Ventilator Waveform Data
Source: Front Med (Lausanne). 2020 Aug 21;7:541. doi: 10.3389/fmed.2020.00541 (PMC7472529; doi:10.3389/fmed.2020.00541)
Supplement: Supplementary file 1 [file Data_Sheet_1.docx]

Electronic Supplemental Material

Table of Contents

[1. Automatic detection of patient-ventilator asynchrony 1](#_Toc37592599)

[2. Calculation of plateau pressure and quasi-static lung compliance 2](#_Toc37592600)

[3. Calculation of work of breathing 3](#_Toc37592601)

# 1. Automatic detection of patient-ventilator asynchrony

In this study, we considered two most frequently observed types of patient-ventilator asynchrony (PVA), i.e. double triggering (DT) and ineffective inspiratory effort during expiration (IEE). An interpretable deep learning approach was developed for their detection.

1.1 Data collection and annotation

We collected high-granularity ventilator waveform data from 181 invasive mechanical ventilated patients in the ICUs of Sir Run Run Shaw Hospital of Zhejiang University using a ventilator information system (RespCare^TM^, ZhiRuiSi, Hangzhou, China) for training and evaluating the deep learning model. All the patients were ventilated using PB840 ventilator (Covidien, U.S.).

The data annotation protocol was reported previously [1]. In brief, a group of junior professionals (respiratory therapists with 3-5 years of clinical experience and biomedical engineers with knowledge of PVA) annotate the waveforms using a self-developed annotation tool. A group of senior professionals (respiratory therapists with >10 years of clinical experience) review the annotations. The cycles were annotated as “DT”, “IEE”, or “Others”. As individual models were developed for different ventilation modes, the annotation was carried out separately for pressure control ventilation (PCV), volume control ventilation (VCV) and pressure support ventilation (PSV). We randomly excluded cycles from the majority category (i.e. “Others”) to achieve a balance among different categories. The information of the annotated dataset is given in Table S1. Because PCV is more widely used in the investigated center, the number of annotated cycles for PCV is much higher than that for VCV.

1.2 Model development

The deep learning model was based on a one-dimensional (1D) convolutional neural network (CNN). The architecture of the model is given in Figure S1. It has two channels, which takes the airway pressure and flow waveforms as input, respectively. Each channel extracts the characteristic features from the pressure and flow waveforms, respectively, using a modified 1D-AlexNet model [2]. The outputs from the two channels were concatenated and processed by a global averaging pooling layer, which allows the representation of categories confidence map to interpret the classification results [3]. In the end, a softmax layer was adopted to perform binary classification.

Five independent models were established for detecting different types of PVA under different ventilation modes. They are indicated as H_(PCV, IEE)_, H_(PCV, DT)_, H_(VCV, IEE)_, H_(PSV, IEE)_, and H_(PSV, DT)_. The model for detecting DT under VCV mode was not established due to lack of sufficient data. In the model application, the DT events under VCV mode was detected using the model H_(PCV, DT)_.

1.3 Training and evaluation

The models were trained and evaluated using a 5-fold cross-validation scheme. For each type of PVA under a single ventilation mode, the dataset was split into 5 folds. Four folds were used for training the model, in which 70% were used for learning the weights of neuron connections and 30% were used for optimizing the super parameters of the model. One fold was left for testing. The process was repeated for 5 times to obtain an average performance. The results are shown in Table S2. The interpretation of PVA is illustrated in Figure S3. The best models were adopted for PVA detection in this study.

The models were developed in Python using Keras as the deep learning library. The workstation used for training the models is equipped with Intel Core i7-8700 CPU, 16 GB of memory, and an NVIDIA GeForce GTX 1060 Graphics Processing Unit (GPU).

# 2. Calculation of plateau pressure and quasi-static lung compliance

Both plateau pressure (P_plat_) and quasi-static lung compliance were computed from the high-granularity ventilator waveform data under pressure control (PC) ventilation mode. P_plat_ was estimated from the pressure waveform at the end of inspiration when flow is or is close to zero. The PVA cycles or the cycles showing active exhalation (pressure at the end of inspiration – preset inspiratory pressure > 1 cmH_2_O) were excluded from the calculation.

Hourly quasi-static compliance of the respiratory system (C_qstat_) was computed following the flowchart shown in Figure S2. The whole recording was split into one-hour based episodes. The episode exhibiting severe PVA (AI > 10%) [4] was excluded from the calculation. Static compliance (C_stat_) for each cycle was calculated according to

$C_{stat}=\frac{V_{T}}{P_{plat}-PEEP}$ .

The mean C_stat_ (C_stat-m_) was obtained using a 5-minute moving averaging window. The lowest C_stat-m_ in a one-hour episode was selected to represent the quasi-static compliance (C_qstat_) of the hour. The selection is based on the consideration that the spontaneous breathing effort is likely to cause an increased V_T_ and thus elevated compliance. Therefore, the lowest compliance may approach the real static compliance.

The missing C_qstat_ values were imputed using an autoregressive moving average (ARMA) process model. The ARMA model assumes that the value in a stationary stochastic process at time *t* can be represented as the weighted sum of *q* random variables and *p* weighted sum of previous observations, according to equ. (2)

$x_{t}=\alpha_{1}x_{t-1}+\alpha_{2}x_{t-2}+\cdots+\alpha_{p}x_{t-p}+\beta_{1}z_{t-1}+\beta_{2}z_{t-2}+\cdots+\beta_{q}z_{t-q}$ ,

where *x* and *z* indicate observations and random variables, respectively. To apply the model for the imputation problem, first, we tested the stationarity of the hourly C_qstat_ time series using KPSS test [5]. If the stationarity was not satisfied, we iteratively differentiated the series and performed the test until the results becomes stationary. Second, the coefficients $\alpha_{i}$ and $\beta_{i}$ were determined based on the observed data using maximum likelihood estimation. The orders *p* and *q* were determined by Akaike information criterion and Bayesian information criterion. Finally, we imputed the missing values by forecasting them according to the estimated ARMA model. The imputation was implemented using Matlab R2019a (Mathworks Inc., U.S).

# 3. Calculation of work of breathing

Inspiratory work of breathing (WOB) was calculated from the pressure-volume loop as shown in Figure S4. It includes both the elastic work and the resistive work. It was averaged hourly for the analysis in this study.

References

[1] L. Zhang, K. Mao, K. Duan, S. Fang, Y. Lu, Q. Gong, F. Lu, Y. Jiang, L. Jiang, W. Fang, X. Zhou, J. Wang, L. Fang, H. Ge, Q. Pan, Detection of patient-ventilator asynchrony from mechanical ventilation waveforms using a two-layer long short-term memory neural network, Comput. Biol. Med., 120 (2020) 103721.

[2] A. Krizhevsky, I. Sutskever, G.E. Hinton, ImageNet Classification with Deep Convolutional Neural Networks, neural information processing systems, 2012, pp. 1097-1105.

[3] B. Zhou, A. Khosla, A. Lapedriza, A. Oliva, A. Torralba, Learning Deep Features for Discriminative Localization, 2016 IEEE Conference on Computer Vision and Pattern Recognition (CVPR), 2016, pp. 2921-2929.

[4] A.W. Thille, P. Rodriguez, B. Cabello, F. Lellouche, L. Brochard, Patient-ventilator asynchrony during assisted mechanical ventilation, Intensive Care Med., 32 (2006) 1515-1522.

[5] K. Denis, C.B.P. Peter, S. Peter, Testing the Null Hypothesis of Stationarity Against the Alternative of a Unit Root: How Sure Are We That Economic Time Series Have a Unit Root?, Cowles Foundation for Research in Economics, Yale University, 1991.

Table S1 The information about the annotated PVA dataset

| Ventilation Mode | Asynchrony  Type | Amount of Data  (Async/Non-Async) |
| --- | --- | --- |
| PCV | IEE | 26480/27971 |
|  | DT | 9915/10297 |
| VCV | IEE | 4656/4713 |
|  | DT | 117/120 |
| PSV | IEE | 14773/14621 |
|  | DT | 559/580 |

Table S2 The performance of the PVA detection models under different ventilation modes

|  | PVA Type | | | | | |
| --- | --- | --- | --- | --- | --- | --- |
|  | IEE | | | DT | | |
| Ventilation mode | Accuracy | Sensitivity | Specificity | Accuracy | Sensitivity | Specificity |
| PCV | 0.985  ±0.002 | 0.987  ±0.003 | 0.984  ±0.003 | 0.988  ±0.001 | 0.991  ±0.002 | 0.985  ±0.002 |
| VCV | 0.973  ±0.003 | 0.978  ±0.004 | 0.968  ±0.006 | / | / | / |
| PSV | 0.970  ±0.001 | 0.974  ±0.005 | 0.966  ±0.004 | 0.955  ±0.009 | 0.977  ±0.013 | 0.935  ±0.025 |


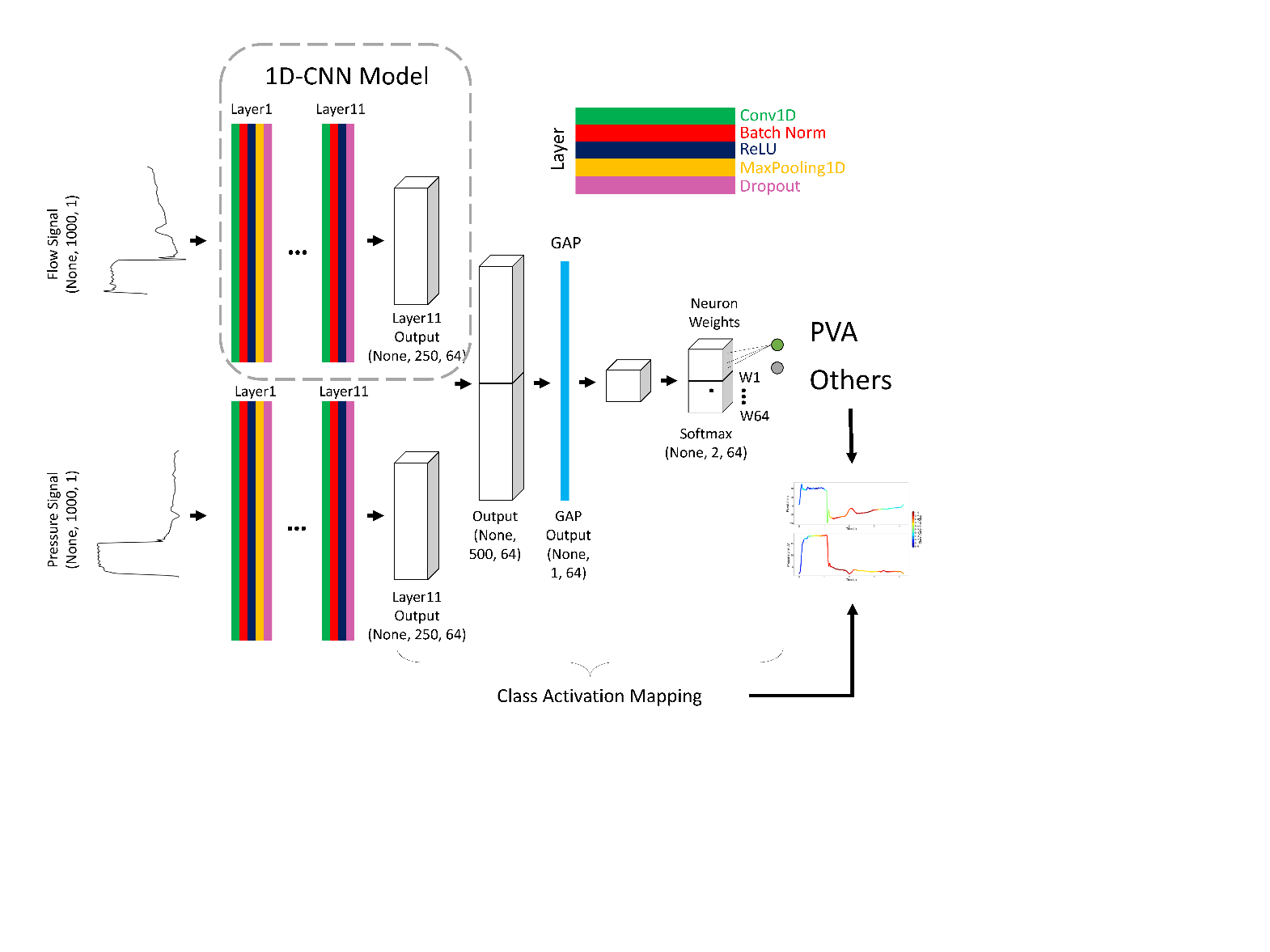


Figure S1 Architecture of the interpretable deep learning model

Figure S2 Flowchart of the quasi static compliance calculation


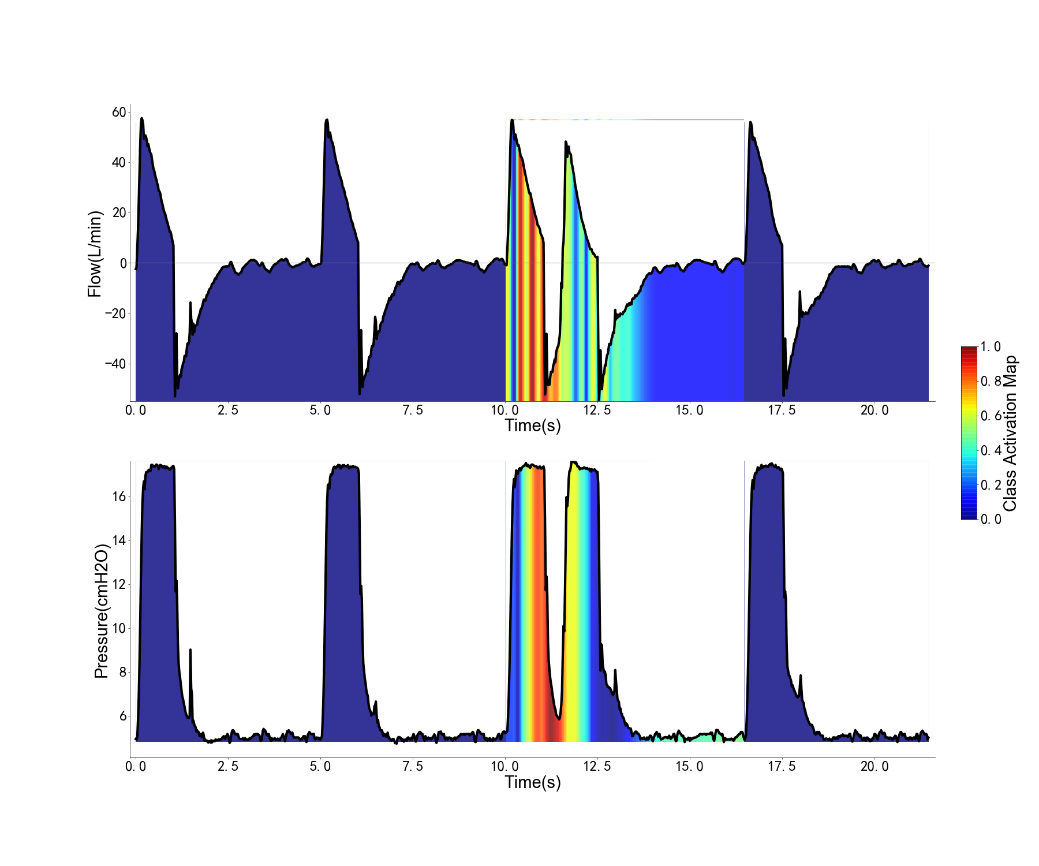


(a)


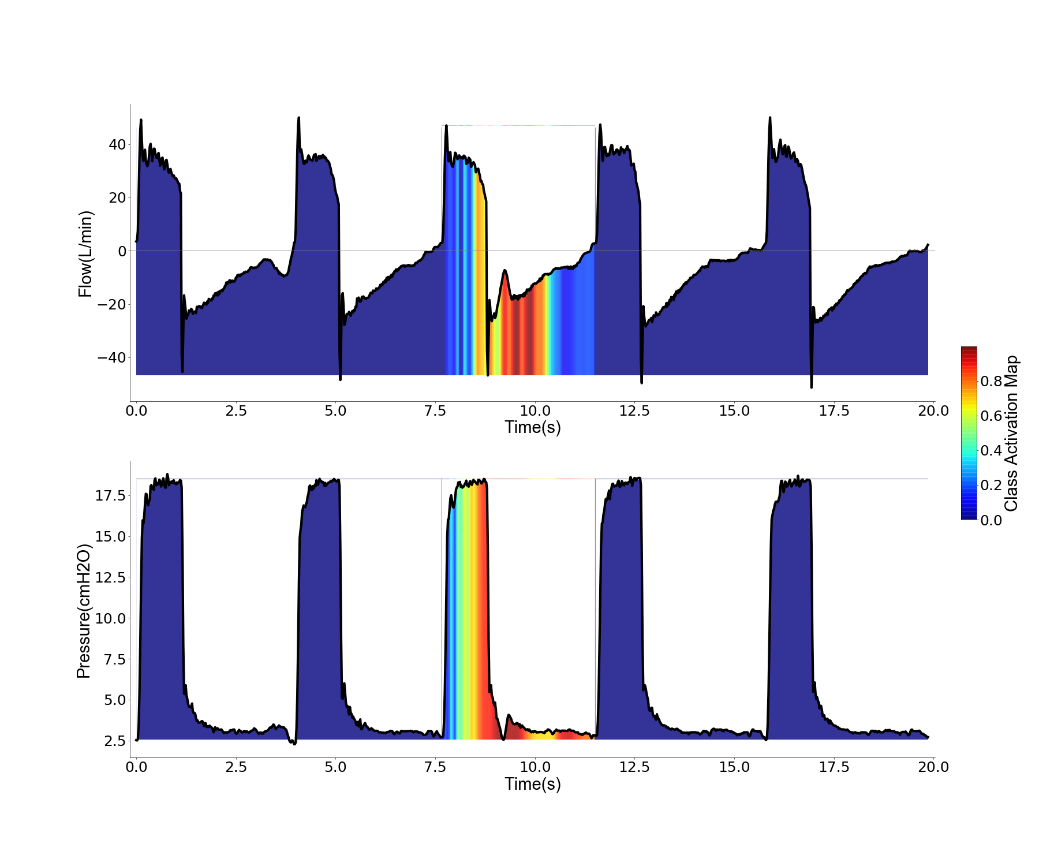


(b)

Figure S3 Interpretation of the cycles classified as PVA. (a) DT cycle (b) IEE cycle. The section that contributes mostly to the PVA determination is highlighted.


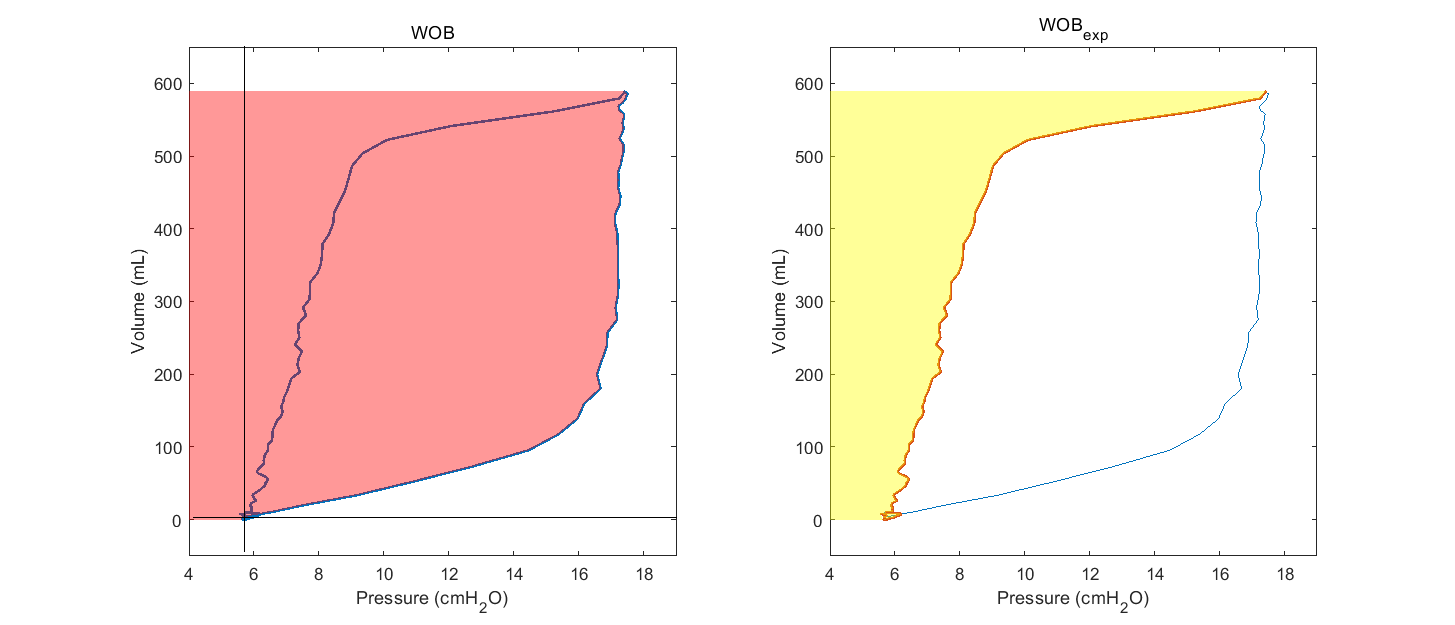


Figure S4 Illustration of the working of breathing calculation
